# Study on the Compressive Strength of Alkali Activated Fly Ash and Slag under the Different Silicate Structure

**DOI:** 10.3390/ma14092227

**Published:** 2021-04-26

**Authors:** Zhipu Wang, Rezeye Rehemituli, Xiaolei Zhang

**Affiliations:** Faculty of Engineering, China University of Petroleum-Beijing, Karamay 834000, China; rezeye@cupk.edu.cn (R.R.); zhangxiaolei@cup.edu.cn (X.Z.)

**Keywords:** alkali-activated materials, silicate structure of waterglass, amorphous gel content, compressive strength

## Abstract

Due to its high activation efficiency, waterglass has been widely used for alkali activations in geopolymer. In this study, the n(SiO_2_)/n(Na_2_O) (Ms) of waterglass was selected as the variable to investigate the role of the silicate structure on the mechanical properties of harden pastes. Ms was changed by the addition of NaOH to obtain the different silicate group, structure and experiments were performed by employing the liquid-sate ^29^Si nuclear magnetic resonance (NMR), Fourier transform infrared spectroscopy (FTIR), dynamic light scattering (DLS) and gel permeation chromatography (GPC) techniques. Furthermore, selected dissolution, scanning electron microscope (SEM-EDX), X-ray photoelectron spectroscopy (XPS) and FTIR experiments were used to measure the development of the amorphous gel and other materials with different curing condition. Results show that silicate structure of the waterglass was changed via the Si-ONa^+^ formation and the electric charge effect of Na^+^. Under the lower Ms waterglass, the Q^0^, Q^1^ and Q_C_^2^ structure reverted to the main structure of the silicate group, which was kind of lower seize, molecule weight, linear or circular chain lower geopolymerization degree silicon structure. It would accelerate the geopolymerization speed of prepolymer formation. In addition, higher activity degree of Q^0^ and Q^1^ were useful to increase the formation amount of the gel structure with a low Si/Al ratio and size. Thus, silicate structure of waterglass controls the amorphous gel properties to adjust the compressive strength of alkali-activated materials.

## 1. Introduction

Recently, emissions of Portland cement into the environment have become a major impediment in the development of green construction materials. Alkali-activated materials, which are inorganic polymer, are defined as three-dimensional aluminosilicate amorphous structures resulting from the reaction of activated aluminum silicate materials and an alkaline activator, and these have become excellent substitutes to cement materials [1,2,3]. In addition to the excellent mechanical properties of harden pastes, alkalai-activated materials also have excellent durability [4,5], fire and acid resistance [6,7] and low energy consumption of during their production [8]. Due to the excellent properties of geopolymer, they have been extensively used on an industrial scale [8].

As an important component of alklai-activated materials, alkaline activators solutions can substantially influence the properties of fresh and hardened pastes. Due to the high activation efficiency, waterglass has been widely used for alkali activations in geopolymer. However, the complex composition of the waterglass solution makes the adjustment and control of geopolymer properties difficult. Therefore, two direction of research have emerged in this respect: (1) reaction mechanism: oligomers (Si [OH]_4_ and Al[OH]_4_^−^), which are the original raw materials of producing, are easily affect by the dissolution and concentration of active Si and Al components. [9] Based on previous investigations [10,11,12,13,14,15], waterglass can substantially accelerate the geopolymerization of oligomers; this is because of the nature of the silicate (Q^n^) group in the solution, which plays an important role in the geopolymerization and production of an amorphous gel and determines the mechanical properties, durability and fire resistance. (2) Silicate structure of waterglass solution: FTIR [16], Raman spectroscopy [17] and ^29^Si NMR [18] experiments were used to obtain the information of structure of waterglass solution. Vidal [19] found the Q^0^, Q^1^, Q^2^, Q^3^ and Q^4^ species in the silicate solution. Furthermore, the Si/M molar ratio is a crucial parameter that affects the nature of silicate, and an important change point was approximately 1.0. Hunt [20], Aguiar [21] and Lucas [22] thought that the higher-order Q^4^, Q^3^ and Q^2^ transform to lower-order (Q^1^ and Q^0^ because of the breaking of the Si-O-Si bonds to produce Si-O- groups. 

However, these investigations only focused on the silicate structure of waterglass. The effects of the silicate structure of waterglass on the mechanical properties of geopolymer hardened pastes, especially on the relationship between the properties of amorphous gel and the compressive strength, have not yet been investigated in detail. Therefore, in this study, the structure of waterglass with SiO_2_/Na_2_O molar ratio (Ms) was systematically measured by performing the liquid ^29^Si NMR, Fourier transform infrared spectrometer (FTIR), dynamic light scattering (DLS) and gel permeation chromatography (GPC) experiments. Furthermore, in order to accurately investigate the effect of waterglass with different Ms on the compressive strength, sealed and standard curing conditions were employed in this study. SEM-EDS and XPS analyses were used to investigate the development of the amorphous gel, and selected dissolution in acid was used to assess the gel content. Based on these measurements, the effect of the structure of the silicate group on the mechanical properties could be explained comprehensively to provide the development on the adjustment of geopolymer.

## 2. Materials and Methods

### 2.1. Materials 

#### 2.1.1. Binder Materials

**Figure 1:**
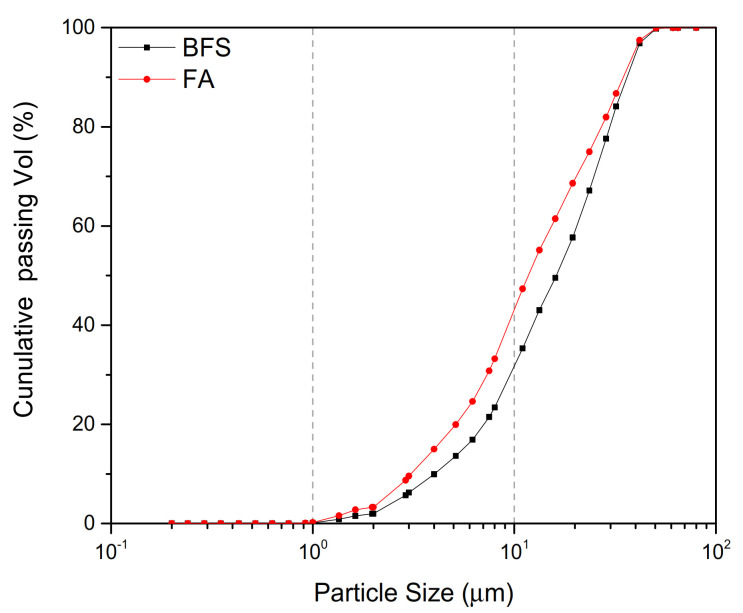
Particle size distribution of raw materials.

In this study, in order to improve the early activity of fly ash, slag was added into the binder materials. Thus, fly ash (abbreviated as FA, being from Da Tang Tongzhou Technology Co., Beijing, Ltd. China) and blast furnace slag (abbreviated as BFS, providing by Capital Iron and Steel Company in Beijing, China) were used as the aluminosilicate binder materials in this paper. The properties (chemical composition, fineness and density) of binder materials are listed in Table 1 and Figure 1 shows the particle size distribution of FA and BFS.

#### 2.1.2. Alkaline Activators

In this study, NaOH (A.R., >99% pure, Beijing Chemical Works, China) was used to adjust the Ms of waterglass Ms = 2.42, providing by Hong-Xing Ltd. Beijing, China) attributing to the high pH environment and did not add any further chemical elements in the systems. As reported by Vidal [19] waterglass samples with three Ms ratios (Ms = 2.4, 2.0 and 1.5) were prepared by the addition of NaOH. These were stored in a container for 24 h and then returned to the 25 °C. The amount of added NaOH is shown in Table 2.

### 2.2. Experimental Method

#### 2.2.1. Properties of Alkaline Activators 

**Figure 2:**
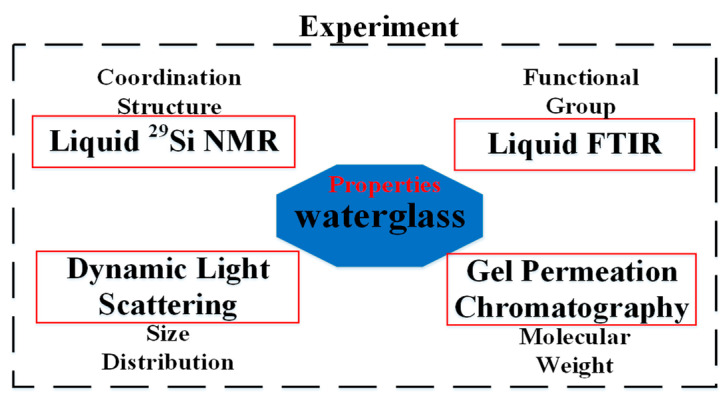
Properties of waterglass.

The properties of waterglass were measured by the following experiments, as shown in Figure 2. Liquid-state ^29^Si NMR, liquid-state FTIR, DLS and GPC analyses were performed to investigate the coordination structure, functional groups, size distribution and molecular weight of the silicate group in the waterglass. These specific analyses are discussed in the following parts:

##### Liquid-State ^29^Si NMR 

Bruker Avance III HD 700 MHz (Bruker Daltonics, Billerica, Germany) was used to record the liquid-state ^29^Si NMR spectra to acquire the information of coordination structure of silicate at room temperature. The experiment date was acquired by the BBO probes. In this study, undiluted waterglass activators were dissolved in heavy water (D_2_O) for the measurements. 

##### Liquid-State FTIR

A Nicolst Is10 Spectrometer (Thermo Fisher Scientific, Waltham, MA, USA) was used with 50 μL aliquots of the sample liquid to measure the chemical functional groups of waterglass at room temperature. A total of 256 scans per sample with different Si/Na molar ratios were collected at a resolution of 4.0 cm^−1^, over 4000–500 cm^−1^.

##### Dynamic Light Scattering (DLS) of Alkaline Activator Solution 

In the present work, the size of flocculated waterglass structure was obtained by the DLS experiment (Horiba SZ-100Z, HORIBA JY, Paris, France). Waterglass samples with three Ms ratios were added into the 10 mm × 10 mm optical silica cuvettes to measure under 826.3 nm laser irradiation. 

##### Gel Permeation Chromatography (GPC) of Alkaline Activators Solution

A gel permeation chromatography analyzer (1260 Infinity Ⅱ HT GPC, Agilent, Santa Clara, CA, USA) was used to investigate the molecular weight distribution of waterglass as follows: first, 0.1 g of samples (accurate to 0.001 g) was accurately weighed and dissolved into the mobile phase, i.e., 1 g of distilled water; then samples were added into the analyzer and finally, the separation time and peak area of components were recorded.

#### 2.2.2. Preparation and Properties of Geopolymer Pastes 

##### Preparation of Geopolymer Pastes

The geopolymer pastes were prepared according to GB/T8077-2012 [23] (Table 3) as follows: first, the FA/BFS powder was added in the mixer. then, the alkaline activations were put into the mixer and finally the mixture was stirred. The well-mixed fresh geopolymer pastes were cured under the curing condition. 

##### Curing Conditions 

To avoid the impact of curing conditions on the samples with different Ms, two curing conditions (sealed curing condition, 25 °C, isolated air, C-1; standard curing condition, 25 °C, 95 % RH, C-2) were employed to investigate the effect of Ms on the compressive strength of samples until testing time. In this study, samples under the sealed curing conditions were stored in plastic boxes wrapped with plastic film to keep out the air. 

#### 2.2.3. Properties of Hardened Geopolymer Pastes

Compressive strength of hardened geopolymer pastes with different curing conditions.

The compressive strength of hardened samples was investigated using CTM 200 kN testing machine with a loading rate of 2.4 kN/s. Three samples of 3 d, 7 d and 28 d were tested and the average value was recorded. 

#### 2.2.4. Microstructure

##### SEM

The morphology was determined using a scanning electron microscopy (SEM, JEOL JSM-7001F, Japan) with energy dispersive X-Ray Spectroscopy (EDS). Samples polished in the Cu paper and then coated with the gold.

##### XPS 

Changes in binding energies of Si-O-Si of the geopolymer were investigated by X-ray photoelectron spectroscopy (XPS). An Escalab 250Xi instrument (Thermo Fisher Scientific, Waltham, MA, USA) with Al Ka radiation was used. 

#### 2.2.5. Selective Dissolution 

According to previous researchers, C-S-H and C(N)-A-S-H are known to be the reaction products in fly ash/metakaolin geopolymer [24,25,26]. The independent analysis of C-S-H and C(N)-A-S-H gel was difficult because of their co-existence in the microstructure, especially in the early-age samples. Selective dissolving has been used to investigate the co-existing of C-S-H and C(N)-A-S-H of geopolymer pastes, through salicylic acid (SAM)/HCl extraction of the geopolymer powder sample. For geopolymer pastes, SAM extraction involves the dissolution of calcium silicate hydrate and not of unreacted fly ash, slag or geopolymer [24,25]. Similarly, HCl induces the dissolution of the chief reaction products of geopolymer, i.e., the aluminosilicate gel and zeolites. However, calcium silicate hydrate decomposed into silica gel under HCl extraction conditions. Therefore, this study investigates the C(N)-A-S-H gel via the two steps: SAM-HCl experiments. 

SAM extraction: first, the geopolymerization of samples was stopped by using methanol; then, samples were dried at 60 °C and finally 1 g of the geopolymer powder sample was added to a solution containing 4 g of salicylic acid and mixed in 60 mL of methanol. The mixture was stirred for 2 h, and the suspension was vacuum filtered with a filter having a 0.2 μm pore size. Furthermore, the insoluble residue was washed with methanol and stored in a vacuum desiccator. 

HCl extraction: first, the geopolymerization of samples was stopped by using methanol; then samples were dried at 60 °C, and 1 g of the activated geopolymer pastes after SAM extraction was added into 250 mL HCl (1:20, volume). The mixture was stirred for 3 h, followed by filtration. The insoluble residue was washed with deionized water several times to realize a pH = 7 of water, dried at 100 °C for 24 h and stored in a vacuum desiccator.

## 3. Results

### 3.1. Inluence of Ms on the Properties of Waterglass 

#### 3.1.1. Composition of Waterglass with Different Ms

**Figure 3:**
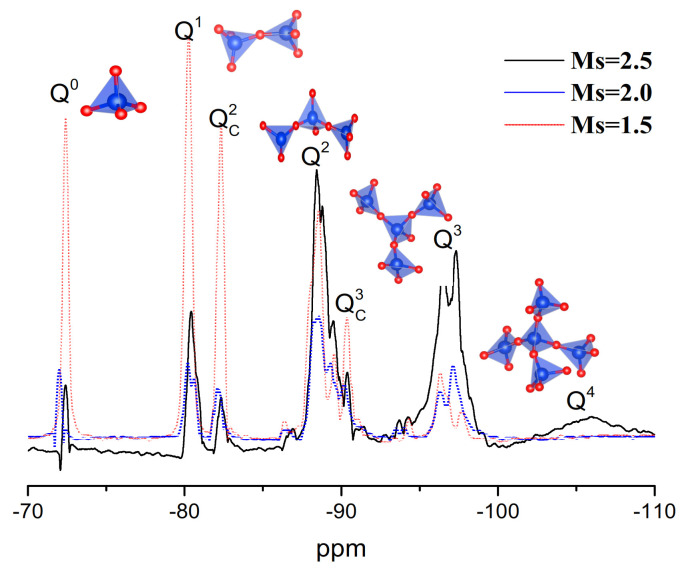
29Si NMR results for silicon in the alkaline activator solution.

**Figure 4:**
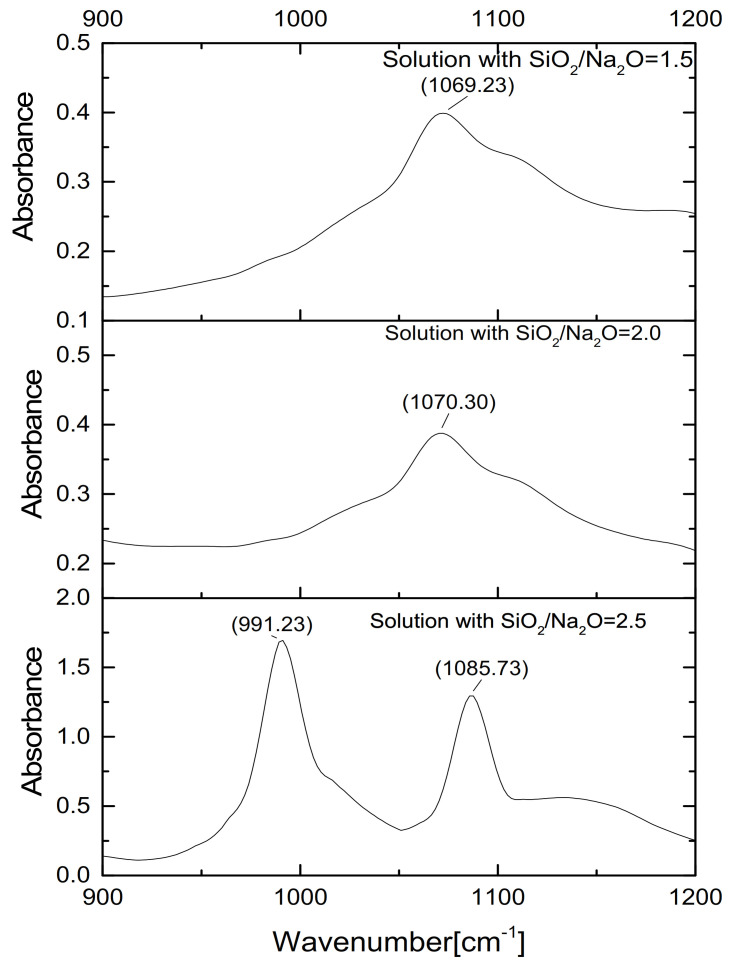
FTIR spectra of alkaline activators with different Ms.

29Si liquid-state NMR results for waterglass with the different Ms are shown in Figure 3. Seven main signals are evident at −72.4, −80.2, −82.3, −88.4, −90.4, −96.5 and −106.9 ppm corresponding, respectively, to Q^0^, Q^1^, Q^2^, Q_c_^2^, Q3, Q_c_^3^ and a low-intensity peak Q^4^ [27,28,29]. It clearly seen that with the decreasing of Ms of samples, the main peaks shift to the left centering to Q^0^, Q^1^ and Q_c_^2^. This result was the same as those of Hunt [20], Aguiar [21] and Lucas [22] investigation. The Q^0^, Q^1^ and Q_c_^2^ silicate structure mainly linear or circular chain lower-order structures, which could indicate that the bond of condensed silicon species was destroyed, result in the depolymerization. Furthermore, the NBO (Si-ONa^+^) group was formed by the combination of positively charge Na+ with small particles and the destruction of the Si–O–Si group. This result agreed with the results for strength and position of the Si–O–Si peak (108.73 cm^−1^), as shown in Figure 4.

#### 3.1.2. Properties of Waterglass with Different Ms

In order to study the effect of the silicon structure on the properties of the waterglass solution, the particle size distribution and molecular weight distribution were investigated.

**Figure 5:**
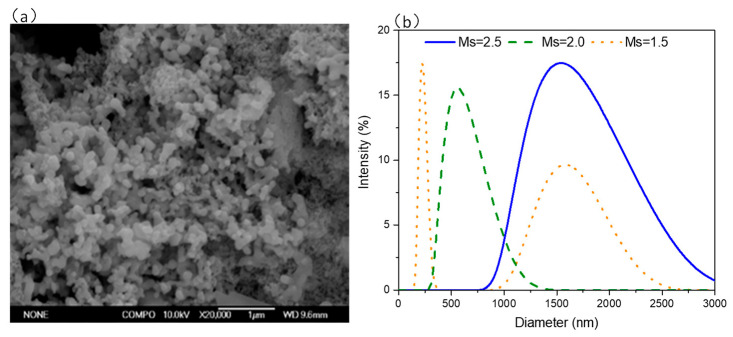
(**a**) Morphology and (**b**) DLS (Dynamic light scattering) spectra of alkaline activators.

Figure 5 shows the results of DLS measurement of waterglass with different Ms. The intensity is used as the index to evaluate the particle size distribution of samples. The mean hydrodynamic radius for the SiO_2_/Na_2_O ratio of 2.5 is 1514.1 nm. This value was larger than the size of a single particle (about 1–5 nm) [30], indicating the aggregation of silicate particles, as confirmed by the SEM image of waterglass in Figure 5a. With the decreasing SiO_2_/Na_2_O ratio of 2.0 and 1.5, the mean hydrodynamic radius became 560.2 nm and 246.2 nm, respectively, which are lower than those corresponding to the ratio of 2.5.

**Figure 6:**
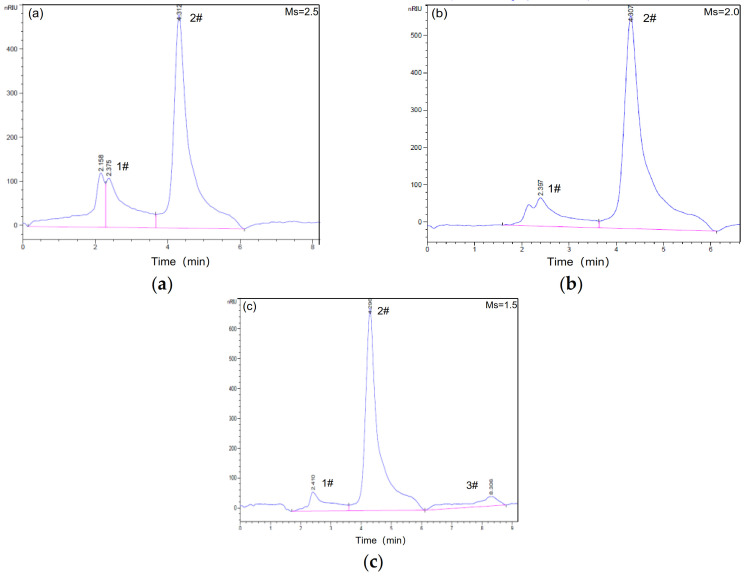
GPC spectra of waterglass with different Ms: (**a**) Ms = 2.5, (**b**) Ms = 2.0, (**c**) Ms = 1.5.

Figure 6 shows the molecular weight distribution of the alkaline activators determined by the GPC measurements. Three peaks can be seen in the curves, including a primary peak at approximately 4.29–4.31 min (2#), a second peak at 2.19–2.41 min (1#) and a third peak at 8.31 min (3#), which were assigned to the three types of molecular structures in the waterglass. With decreasing Ms, the characteristic peak strength and area of the second and third peaks show a significant increase. However, the trend for the primary peak is opposite. It well known that the larger molecular weight of samples could not through the small pore structure of gel column to reduce outflow time, which resulting in the smaller retention time of molecular [31]. Therefore, the molecule weight of samples obviously decreases for Ms < 1.5.

The experimental results show that for Ms = 1.5, i.e., waterglass with linear lower-order structure, a smaller size and lower molecule weight were observed. This may be associated with the electric charge effect of Na^+^ and formation of Si-ONa^+^. For the lower Ms waterglass solutions, the free Na^+^ easily absorbed on the surface of silicate group, thus increasing repulsion among the group. The formation of linear and circular chain possibly decreases the probability of agglomerate due to the reduction in the number of contact point on the surface. Thus, it would efficiently destroy the agglomerate structure of waterglass with Ms = 2.5 resulting to the decreasing of size and molecular weight of waterglass.

### 3.2. Mechanical Properties of Geopolymer Hardened Pastes with Different Ms

**Figure 7:**
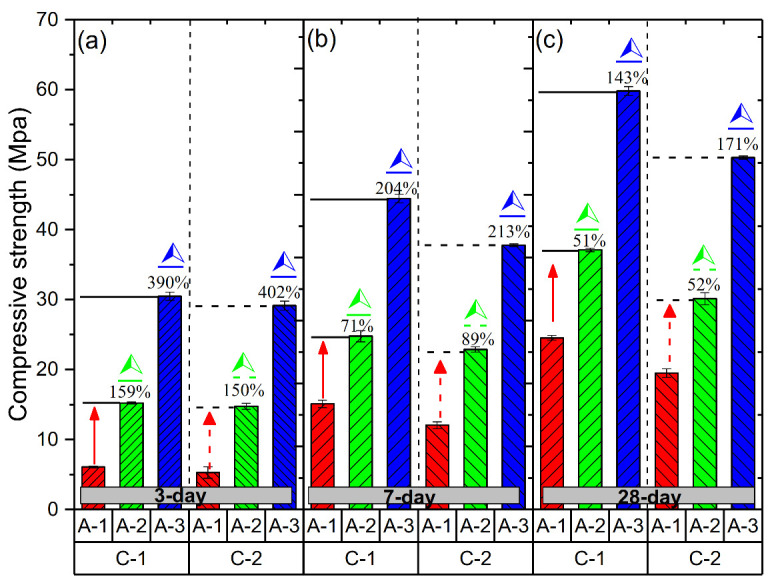
Compressive strength of samples with different alkaline activators under two curing conditions: (**a**) 3 days, (**b**) 7 days and (**c**) 28 days. (⮙ indicates the increases value of compressive strength compared with A-1# samples).

The compressive strength results for A-1#, A-2# and A-3# samples that were cured for up to 3, 7 and 28 days, respectively, at the different curing C-1 and C-2 condition are shown in Figure 7. All showed the same tendency of change in the compressive strength under the different curing conditions, which indicated that this phenomenon was influenced by the Ms. It is clearly seen that A-3# samples had the highest compressive strength under the C-1 and C-2 curing conditions at 3 days. Comparing with A-1# samples, the relative increasing strength ((Compressive strength of A-2#/A-3#− Compressive strength of A-1#) ÷ strength of A-1#0) × 100%, at same curing age) of A-3# samples reach up to 390%. In addition, compressive strength of A-2# also has 129% growth rate. As shown in Figure 7, the rate of increase in the compressive strength significantly decreases with time. It is clear that A-3# samples with Ms = 1.5 show a high rate of increase in the compressive strength at all curing conditions: nearly 390%, 204% and 143% at 3, 7 and 28 days, respectively. Furthermore, the A-3# samples show the highest strength values at 28 days (59.8 MPa under C-1 and 50.3 MPa under C-2), as shown in Figure 7c.

### 3.3. Development of Amorphous Gel with the Different Ms

#### SEM-EDS

**Figure 8:**
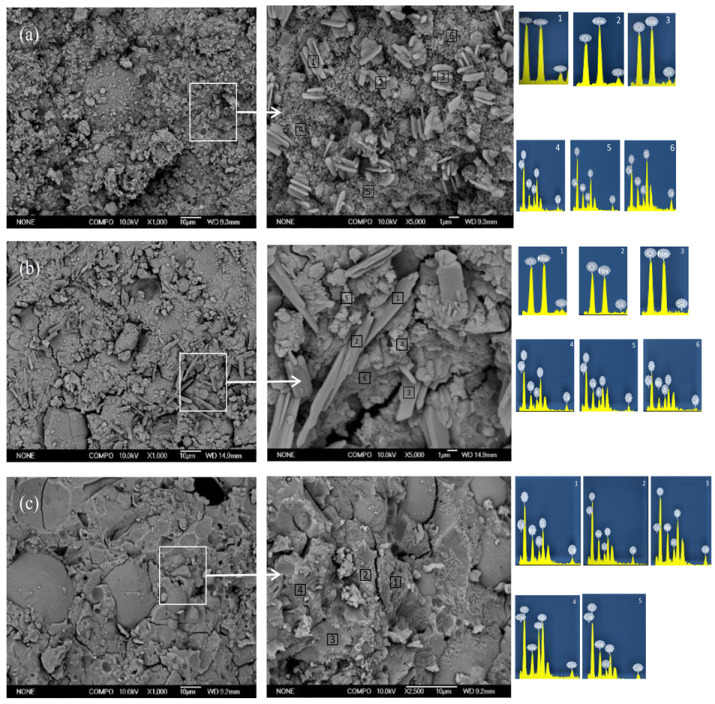
SEM images of samples with different Ms under the C-1 curing conditions after 3 days: (**a**) A-1, (**b**) A-2 and (**c**) A-3.

Morphology changes of the geopolymer hardened pastes were investigated by performing the SEM-EDX analysis. Figure 8 shows the morphologies of samples with different Ms after the 3-day curing; the gel structure and particles existed in the samples. Specifically, flaky particles and gel structure are shown in Figure 8a. According to the EDS experimental results (Table 4), Na, O and Si were the main elements of flaky particles with Na/Si molar ratio of 2, which indicates that these could be unreacted waterglass. According to the results of previous investigations [16,17,18], the number of unreacted waterglass particles of higher Ms ratio. Furthermore, the C(N)-A-S-H gel is observed in Figure 8b (according to the Table 4). It is clearly seen that unreacted alkaline activators particle do not exist in the A-3# samples. Comparisons with A-1 and A-2 that the geopolymer hardened pastes gradually become denser due to the formation of the amorphous gel in the A-3# samples. Furthermore, the Si/Al ratio of the gel with different Ms ratios is investigated though SEM-EDX, as shown in Table 4. The gel of A-3# samples had the lowest Si/Al ratio, which may be related to the charge balance of alkali metal ions.

**Figure 9:**
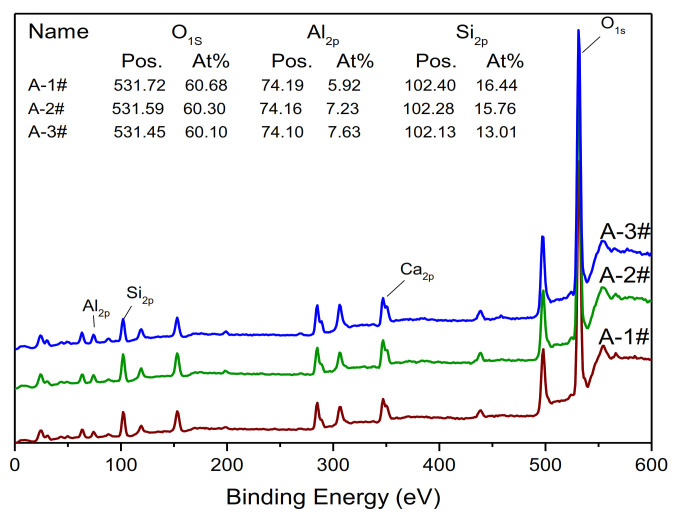
XPS curves of samples with different Ms under the C-1 curing conditions.

**Figure 10:**
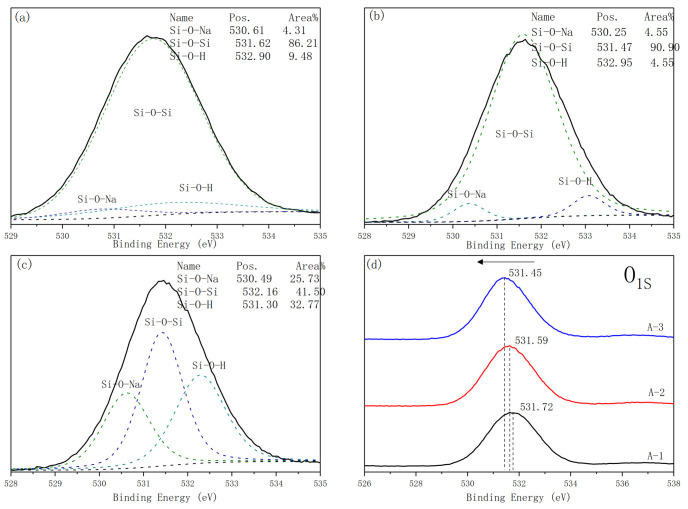
Fitting of O_1S_ line taken from: (**a**) A-1, (**b**) A-2, (**c**) A-3 and (**d**) all samples curing C-1 conditions.

As is well known, the properties of the amorphous gel are dependent on the chemical bonds between Si, Al and O. The XPS results are shown in Figure 9 and Figure 10. In addition to expected Si and Al peaks, the O peak is observed in all the samples. From Figure 6, it can be seen that the increase in Ms leads to a slight increase in the Si 2P binding energy, from 102.13 to 102.40 eV. Furthermore, the Al2p binding energy decreases from 74.19 to 74.10 eV between A-1 and A-3. Generally, the tetrahedral aluminum has a lower binding energy than the octahedral aluminum:73.2–74.35 and 74.1–75.0 eV [32]. Therefore, the structure of tetrahedral aluminum did not change with the change in Ms.

Oxygen has a major role in the formation of the geopolymer. As Figure 10 shows, an unsymmetrical oxygen peak exists, which indicates the presence of different chemical states of oxygen. According to results of previous studies [33,34,35,36], Si–O–Si, Si–O–Al, Si–O–H and Si–O–Na. Si–O–Si bonds correspond to the silicon skeleton, and the peak ascribed to silanol (Si–OH) bonds is reported to be located at 532–533 eV. In this study, non-bridging oxygen, i.e., in Si–O-Na bonds is present in negligible amounts. It is clear that the percentages of Si–O–Na and Si–O–H bonds increase with decreasing Ms of A-2 and A-3. With the decreases in Ms, the percentage of the Si-O-Na bonds rapidly increases to a maximum value of 25.73% in A-3. It is known that Si–O-Na groups situated at the extreme ends of geopolymer chains are closely related to the size of geopolymer particles [37].

**Figure 11:**
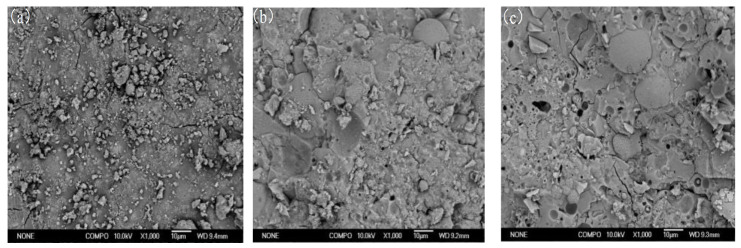
SEM images of samples with different Ms under the C-1 curing conditions after 28-day: (**a**) A-1, (**b**) A-2 and (**c**) A-3.

**Figure 12:**
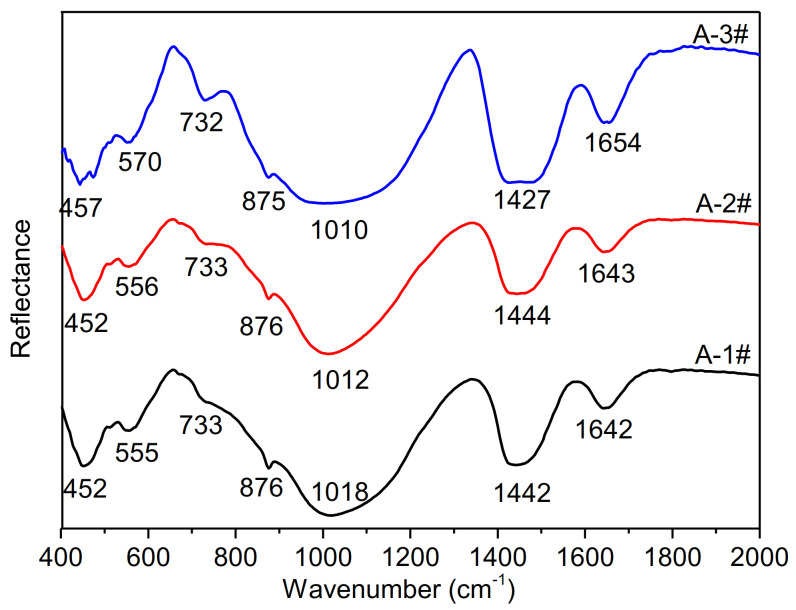
FTIR spectra of samples with different Ms at C-1 curing condition.

**Figure 13:**
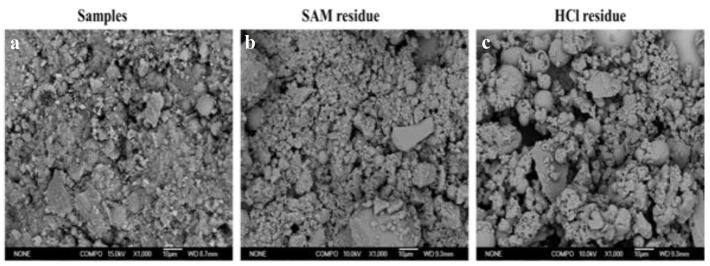
SEM spectra of samples after dissolution with different acids: (**a**) bulk samples, (**b**) samples after SAM, (**c**) samples after HCl.

**Figure 14:**
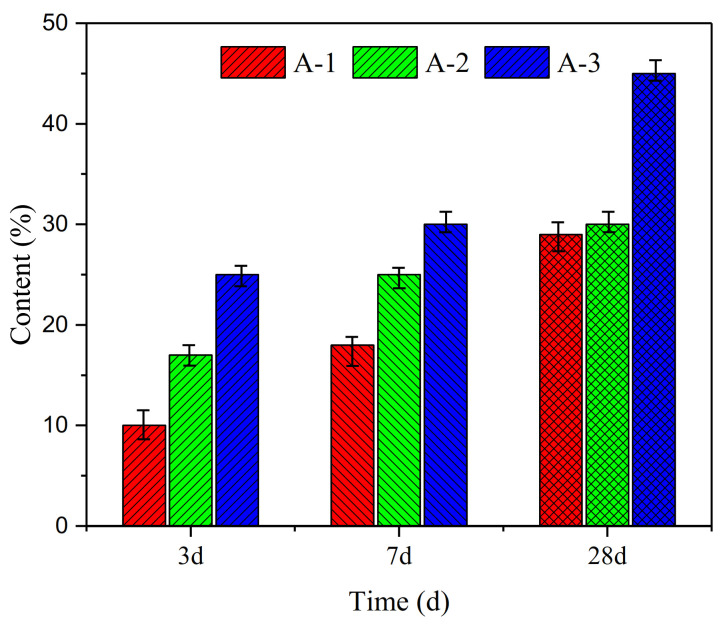
Gel content of samples with different Ms was measured by selective dissolution.

Figure 11 shows the morphology of A-3# samples after the 28 days of curing. With increasing curing time, the compactness of hydration products on the sample particles increased. This might explain why A-3# had the highest compressive strength after up to 28 day. A comparison with A-2# and A-1# shows that the Si-O bending intensity of A-3# decreases because of the higher reaction degree of samples at 28-day, as shown in Figure 12. It is clear that there exists the higher Al–O stretching peak at A-3# than the A-1# and A-2#. In the A-3# samples, the T–O stretching band shifts to lower wavenumbers from 1018 cm^−1^ for Ms = 2.5 to 1010 cm^−1^ for Ms = 1.5, indicating the formation of the Si–O–Al network. This shift is attributed to the formation of weaker Al−O bonds. In addition, the Si–O–T peak with obtuse shape indicates the higher amorphous degree. In order to quantitatively investigate the gel content of samples with different Ms, selective dissolving was employed. Results was shown in Figure 13 and Figure 14.

Based on the investigation of Puligilla [25,26], the method of SAM-HCl selective dissolution was effective to measure the development of the co-existing of C-S-H and N(K)-A-S-H gel in the fly ash—slag beads geopolymer. After the selective dissolution using the acids of SAM and HCl, the morphology of samples is shown in Figure 13. Different acid caused the dissolution of different product components. A comparison with the samples that did not undergo dissolution shows that samples after SAM and HCl dissolution had a low density of structure and highly smooth particles surface, indicating a decrease in the number of reaction product covering the particles. However, most reactions product had been dissolute after the HCl treatment. Therefore, in this study, selective dissolution was adopted to quantitatively investigate the change in the gel for the different Ms. The results of experiment are shown in Figure 14. As shown, samples with Ms = 1.5 have the highest gel content. It also could be seen that the highest amount of gel was obtained in the case of A-3 samples after the 28-day curing when Ms = 1.5 waterglass was used.

### 3.4. Relationship Between Waterglass Properties, Gel Content and Compressive Strength

**Figure 15:**
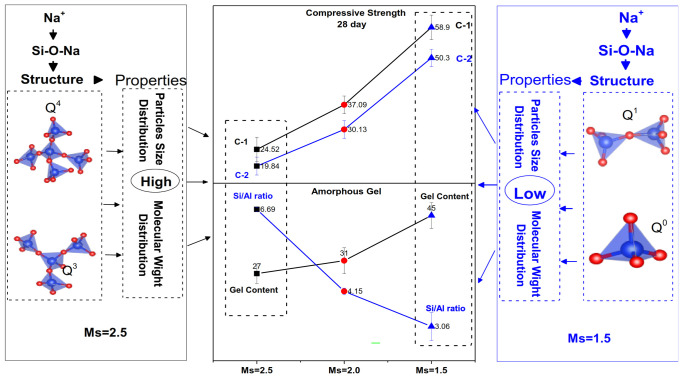
Relationship between waterglass properties, gel content and compressive strength.

Figure 15 shows the linkage between waterglass properties, gel content and compressive strength. The compressive strength of geopolymer hardened pastes was influenced by the amorphous gel content, properties of waterglass and structure of silicate group structure. At Ms = 1.5, increasing the Na^+^ and OH^−^ contents destroys the Si-O-Si to form the Si-O-Na and free Na^+^ ion easily absorbed on the surface of the silicate group, thereby increasing the repulsion between the group. These could be the reasons for the intrinsic properties (in terms of particles size distribution and molecular weight distribution) of the waterglass solution. The lower size and molecular weight may efficiently improve the chances for contact, thereby accelerating the formation of the amorphous gel. Furthermore, a higher Na^+^ content could be helpful for the formation of Si-O-Na resulting in the formation of the amorphous gel with the lower size and Si/Al ratio. Moreover, it improves the density of the geopolymer hardened pastes. Thus, the highest compressive strength of the geopolymer corresponded to Ms = 1.5.

## 4. Discussion

The compressive strength of geopolymer hardened pastes was influenced by the amorphous gel content, properties of waterglass and structure of silicate group structure. Increasing the Na^+^ and OH^−^ contents destroys the Si-O-Si to form the Si-ONa^+^, thereby increasing the repulsion between the group to form the lower size and molecular weight of silicate structure. It may efficiently improve the chances for contact, accelerating the formation of the amorphous gel. Thereby it improves the density of the geopolymer hardened pastes.

## 5. Conclusions

In this study, the effects of different Ms of waterglass on the properties of hardened pastes were studied. The aim of the study was to determine the relationship between the structure of the waterglass, gel content and compressive strength of samples. From the obtained results, the following conclusions can be drawn:

1. With decreasing Ms, branched and agglomerated coordination unit groups of silicon were destroyed with the formation of Si-ONa^+^ and the electric charge effect of Na^+^, which produces the linear and circular-chain silicon structures with lower sizes and molecule weight in the Ms = 1.5 waterglass activator.
2. With decreasing Ms, the compressive strength of geopolymer hardened pastes significantly increases. The highest value 58.9 MPa was obtained for Ms = 1.5 after the 28 days curing.
3. The waterglass with low Ms (Ms = 1.5) results in the linear and circular-chain silicon structures with the lower size and molecule weight, which improve the chances of contact of active component, resulting in the improvement in the formation of the gel structure with low Si/Al ratio and size. This could be the major reason for the highest compressive strength of hardened pastes.

## Figures and Tables

**Table 1:** Properties of raw materials.

	Chemical Composition/wt %									Fineness/μm	Density/gcm^−3^
	CaO	SiO_2_	Al_2_O_3_	MgO	Fe_2_O_3_	SO_3_	Na_2_O	K_2_O	LOI		
FA	5.17	43.57	38.36	0.61	5.39	0.99	0.53	0.81	2.73	10.3	2.20
BFS	40.72	24.96	12.84	0.94	0.53	1.858	0.94	0.34	4.63	11.2	1.96

**Table 2:** Waterglass and NaOH contents and (SiO_2_)/(Na_2_O) ratio (Ms).

	Waterglass/wt%	Sodium Hydroxide/wt%	Ms
AA-1	100	0	2.5
AA-2	95.24	4.76	2.0
AA-3	85.42	14.58	1.5

**Table 3:** Design of geopolymer pastes.

Mix	Mass Ratio				
	Binder		W/B	Alkaline Activators	
	FA	BFS		Ms ^a^	Content ^b^
A-1#	0.7	0.3	0.45	2.5	30%
A-2#	0.7	0.3	0.45	2.0	30%
A-3#	0.7	0.3	0.45	1.5	30%

^a^ The Ms is the SiO_2_/Na_2_O molar ratio in the alkaline activator; ^b^ the content of activators is determined as per the proportion of the activator to binder. When Ms < 1.5, the silicate structures of alkaline activators easily reunite to form the sediment.

**Table 4:** Chemical compositions determined by EDS of crystals in Figure 8.

	Postioion	O/wt%	Na/wt%	Si/wt%	Al/wt%	Ca/wt%	Si/Al
**A-1**	1	63.22	25.72	11.06	/	/	/
	2	62.13	26.71	11.16	/	/	/
	3	58.80	26.90	14.30	/	/	/
	4	71.88	8.02	14.32	2.06	3.72	6.75
	5	70.05	8.82	14.41	2.11	3.61	6.59
	6	70.29	8.16	15.06	2.16	3.88	6.72
**A-2**	1	61.71	25.87	12.43	/	/	/
	2	63.40	26.06	10.54	/	/	/
	3	61.83	27.41	10.76	/	/	/
	4	68.13	9.14	14.35	3.25	5.13	4.25
	5	68.59	8.82	13.82	3.29	5.37	4.05
	6	68.35	9.05	14.20	3.30	5.10	4.15
**A-3**	1	69.91	10.34	11.23	4.45	4.08	2.43
	2	71.89	9.27	10.15	3.38	5.32	2.89
	3	72.06	10.01	9.93	3.28	4.73	2.91
	4	69.33	10.25	12.06	3.37	5.00	3.45
	5	70.73	12.27	10.34	2.73	3.93	3.65

## Data Availability

The data presented in this study are available on request from the corresponding author.
